# Effect of once-only flexible sigmoidoscopy screening on the outcomes of subsequent faecal occult blood test screening

**DOI:** 10.1177/0969141318785654

**Published:** 2018-10-03

**Authors:** Jeremy P Brown, Kate Wooldrage, Ines Kralj-Hans, Suzanne Wright, Amanda J Cross, Wendy S Atkin

**Affiliations:** 1 Cancer Screening and Prevention Research Group, Department of Surgery and Cancer, Imperial College London, London, UK; 2 NHS Cancer Screening Programmes, Sheffield, UK

**Keywords:** Colorectal cancer, flexible sigmoidoscopy, screening programme, faecal occult blood test

## Abstract

**Objective:**

To investigate the outcomes of biennial guaiac faecal occult blood test (gFOBT) screening after once-only flexible sigmoidoscopy (FS) screening.

**Methods:**

Between 1994 and 1999, as part of the UK FS Screening Trial (UKFSST), adults aged 55–64 were randomly allocated to an intervention group (offered FS screening) or a control group (not contacted). From 2006, a subset of UKFSST participants (20,895/44,041 intervention group; 41,497/87,149 control group) were invited to biennial gFOBT screening by the English Bowel Cancer Screening Programme. We analysed gFOBT uptake, test positivity, yield of colorectal cancer (CRC), and positive predictive value (PPV) for CRC, advanced adenomas (AAs), and advanced colorectal neoplasia (ACN: AA/CRC).

**Results:**

Uptake of gFOBT at first invitation was 1.9% lower (65.7% vs. 67.6%, *p* < 0.01) among intervention versus control group participants. Positivity was 0.4% lower (2.0% vs. 2.4%, *p* < 0.01) and CRC yield was 0.08% lower (0.19% vs. 0.27%, *p* = 0.14). PPVs were also lower in the intervention versus control group, at 10.3% vs. 12.3% (*p* = 0.44) for CRC, 22.7% vs. 31.4% (*p* < 0.01) for AA, and 33.0% vs. 43.7% (*p* < 0.01) for ACN. Among those who refused FS (*n* = 5532), gFOBT uptake at first invitation was 47.7%, CRC yield was 0.25%, and PPV for ACN was 46.2%. Among FS attenders (*n* = 15,363), uptake was 72.2%, CRC yield was 0.18%, and PPV for ACN was 27.9%.

**Conclusions:**

Uptake, positivity and PPV of gFOBT screening were reduced following prior offer of FS screening. However, a quarter of FS screened participants receiving a diagnostic examination after positive gFOBT were diagnosed with ACN.

## Introduction

In England, a biennial guaiac faecal occult blood test (gFOBT) screening programme, the Bowel Cancer Screening Programme (BCSP), began in 2006.^1^ In addition, roll-out of a programme of once-only flexible sigmoidoscopy (FS) screening at age 55 started in 2013.^2^

Screening for colorectal cancer (CRC), using either FS or the gFOBT, has been demonstrated to reduce CRC cause-specific mortality in randomized controlled trials.^3–7^ Colonoscopy, FS, gFOBT, and faecal immunochemical tests (FITs) have all been included in various screening programmes.^8^ Each screening modality has a different profile in terms of cost, sensitivity, specificity, and burden on endoscopy services. FS is more sensitive than gFOBT and FIT for distal advanced colorectal neoplasia (ACN).^9–15^ It is the only screening modality that has been shown to reduce CRC incidence in multiple randomized controlled trials.^16,17^ However, a single FS does not offer complete protection against distal CRC. Furthermore, as the maximum reach of FS is the splenic flexure, proximal colonic neoplasia is only detected if the findings at FS warrant further investigation by colonoscopy. Achieving high uptake of either FS or gFOBT screening is challenging.^1,18^ The effect of adding once-only FS to biennial gFOBT screening in England has been modelled, with predictions that it may prevent an additional 10,000 cases of CRC and 2000 deaths by 2030.^2^ The true impact is not known and will depend on the uptake, positivity, and yield of CRC from biennial gFOBT following FS, outcomes which are uncertain.

We investigated the impact of prior FS on the outcomes of biennial gFOBT screening using data from participants of the UK Flexible Sigmoidoscopy Screening Trial (UKFSST) who were invited to gFOBT screening through the English BCSP. While there have been a number of studies examining the performance of FS relative to gFOBT, to our knowledge, this is the only study to investigate the performance of biennial gFOBT screening in patients previously randomized to either screening FS or to no screening.

## Methods

The UKFSST (ISRCTN28352761), a multi-centre randomized controlled trial of once-only FS screening,^3^ has been previously described in detail.^3,17,19^ Ethics approval for the trial was obtained from local research ethics committees. Men and women aged 55–64 were eligible for the trial if they were registered to a participating GP practice and did not meet any of the following exclusions: inability to provide informed consent, severe or terminal disease, history of CRC, adenomas or inflammatory bowel disease, life expectancy less than five years, or sigmoidoscopy or colonoscopy within the previous three years. Individuals who met these eligibility criteria were sent information about CRC and FS, and were asked “If you were invited to have the bowel cancer screening test [FS], would you take up the offer?” Individuals who indicated that they would take up the offer of screening were invited to participate in the trial, and those who agreed to participate and provided written informed consent were randomized in a ratio of 2:1 to either a control group (not contacted), or an intervention group (offered FS). During FS, small polyps were removed by polypectomy. Participants were referred for colonoscopy if polyps met any of the following criteria: diameter 1 cm or larger, three or more adenomas, 20 or more hyperplastic polyps above the distal rectum, tubulovillous or villous histology, severe dysplasia, or malignancy.^19^

As part of the English BCSP, adults aged 60–74 (previously 60–69) registered with a GP practice are invited every two years to complete a gFOBT kit (Hema-Screen®), which includes six windows for two samples from three separate stools. Participants with a positive gFOBT outcome (i.e. definitive abnormal) are referred to a specialist screening practitioner and offered colonoscopy or, in a small proportion of individuals (typically <3%), another diagnostic investigation, such as computed tomography colonography. Individuals with three or more adenomas, or one or more large adenomas (≥1 cm), enter colonoscopic surveillance following polypectomy.^20^ Due to age restrictions on gFOBT screening and an extended, rather than immediate, roll-out of the screening programme, only a subset of English UKFSST participants were invited to gFOBT screening. Some individuals aged 75 or older entered the BCSP, for example, as over-age self-referrals. These individuals were excluded from our analyses, as they are not representative of typical invitees to the screening programme.

To obtain information on the outcomes of gFOBT screening in included individuals, the UKFSST cohort was matched with BCSP data covering the period 1 August 2006 to 3 March 2016. To ensure sufficient follow-up of at least six months post-invitation, only invitation rounds where the gFOBT invitation was sent before 3 September 2015 were included in this analysis.

CRCs were identified either through BCSP data or in cancer registry data within six months of a positive gFOBT. Incorporating cancer registry data allowed identification of CRCs in UKFSST participants who did not attend diagnostic investigation following positive gFOBT, who had a diagnostic investigation performed by a private healthcare provider, or where the diagnostic investigation missed the cancer. Though registry data were available only up until 31 December 2014, rather than to 3 March 2016, we do not expect that this will have affected the results considerably, as in any given year only one or two CRCs not present in the BCSP data were identified in the registries within six months of a positive gFOBT. Dates of death up to 31 December 2015 were obtained from the Office for National Statistics via NHS Digital.

Adenomas were classified as advanced if either endoscopy or histology data indicated a size greater than or equal to 10 mm, if villous or tubulovillous histology was present, or if there was high-grade dysplasia. We categorized patients by their most advanced neoplastic finding. Hence, patients with CRC and advanced adenomas (AAs) were defined as having an outcome of CRC. Colonic findings distal to the splenic flexure were categorized as distal. ACN was defined as an outcome of AA and/or CRC.

Outcomes of gFOBT screening are presented by UKFSST randomization status and by compliance with the offer of FS screening. Uptake at first invitation is defined as the proportion of invitees who were adequately screened by gFOBT (i.e. gFOBT outcome of positive (definitive abnormal) or negative (definitive normal) at first invitation). Uptake of any gFOBT kit is defined as the proportion of invitees who were adequately screened by gFOBT in at least one invitation round.

Test positivity is defined as the proportion of participants adequately screened by gFOBT who had a positive gFOBT outcome. Yield of CRC is defined as the proportion of participants adequately screened by gFOBT who had a positive gFOBT and were diagnosed with cancer. Test positivity and yield of CRC are presented both at first gFOBT screen, and over all observed gFOBT screening rounds (i.e. the proportion of gFOBT screened participants who tested positive in at least one screening round; and the proportion of gFOBT screened participants who were diagnosed with CRC in at least one round).

We also present the positive predictive values (PPVs) for CRC, AAs, and ACN, at first gFOBT screen. These are defined as the proportion of participants attending diagnostic investigation following a positive gFOBT, who were diagnosed with the specified outcome. We excluded registry-only CRC diagnoses from these calculations, as we did not have diagnostic examination information for cancers identified outside the BCSP. In online supplemental tables, we provide PPVs and yield of CRC stratified by gender, as well as the stage distribution of CRCs detected at gFOBT screen. Cancer staging was determined using cancer registry data and data collected by UKFSST trial staff.

*P*-values were calculated using Fisher’s exact test. All analyses were conducted in STATA/IC 13.1.

## Results

**Figure 1.**
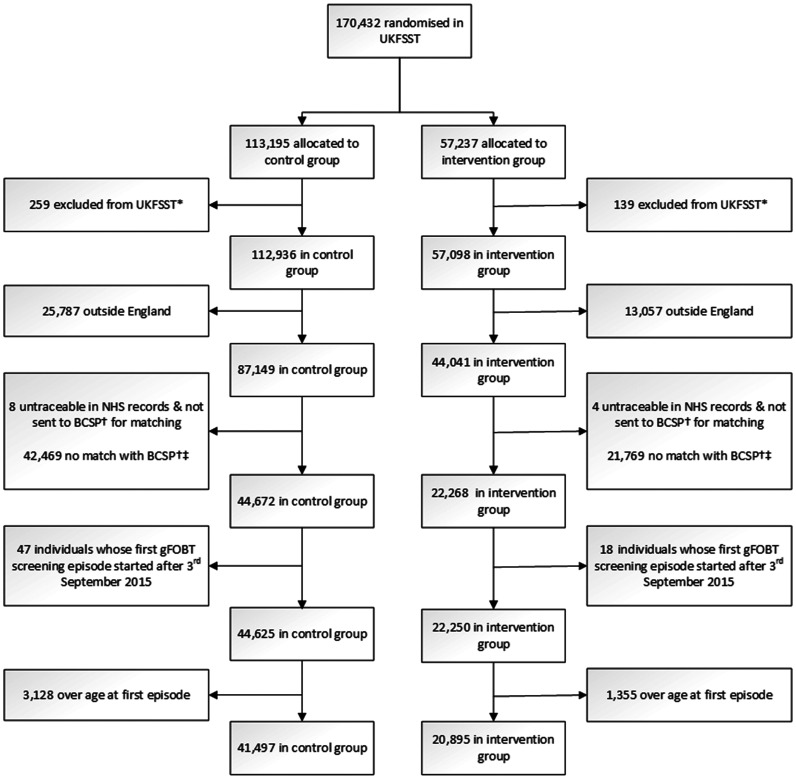
Flow chart of UKFSST participants included in analyses. UKFSST: UK FS screening trial. *Due to pre-randomization events and duplicates.^17^ ^†^BCSP: Bowel Cancer Screening Programme. ^‡^Reasons for no match include death predating BCSP rollout, being above the eligible age for invitation to the BCSP, and emigration.

In the UKFSST, men and women (*n* = 170,432) were randomly allocated to an intervention group *(n* = 57,237) which was offered FS, or a control group (*n* = 113,195) which was not contacted. After exclusions, there were 57,098 individuals in the intervention group and 112,936 individuals in the control group, of whom 44,041 (77.1%) and 87,149 (77.2%), respectively, were recruited in England (Figure 1).

At the initiation of the BCSP, on 1 August 2006, 91.7% (40,389/44,041) of the English UKFSST intervention group and 91.6% (79,870/87,149) of the English UKFSST control group were alive (Table 1). Survival was lower among English UKFSST participants who refused FS (88.1%, 10,866/12,337) than in those who accepted FS (93.1%, 29,523/31,704) or the control group. Furthermore, survival was lower in men (89.4%, 57,522/64,328) than women (93.8%, 62,737/66,862).

**Table 1.** Characteristics of English UKFSST participants.^a^

	Control group	Intervention group		
		Overall	Screened by FS	Refused FS
Characteristics of English UKFSST participants at randomization (*n*=131,190)				
Total in group - *n* (% of cohort)	87,149 (66.4)	44,041 (33.6)	31,704 (24.2)	12,337 (9.4)
Sex - *n* (% of group)				
Men	42,738 (49.0)	21,590 (49.0)	15,994 (49.6)	5596 (45.4)
Women	44,411 (51.0)	22,451 (51.0)	15,710 (50.5)	6741 (54.6)
Age at randomization				
Median	60.0	60.0	60.0	60.1
Interquartile range	57.6–62.5	57.6–62.5	57.6–62.5	57.7–62.5
Characteristics of surviving English UKFSST participants on 1 August 2006 (*n*=120,259)				
Total *- n* (% of group)	79,870 (91.6)	40,389 (91.7)	29,523 (93.1)	10,866 (88.1)
Sex - *n* (%)				
Men	38,237 (47.9)	19,285 (47.8)	14,600 (49.5)	4685 (43.1)
Women	41,633 (52.1)	21,104 (52.3)	14,923 (50.6)	6181 (56.9)
Age - *n* (%)				
60–69	48,426 (60.6)	24,304 (60.2)	17,784 (60.2)	6520 (60.0)
70–79	31,444 (39.4)	16,085 (39.8)	11,739 (39.8)	4346 (40.0)

^a^After exclusions due to pre-randomization events and duplicates.

UKFSST: UK FS screening trial; FS: flexible sigmoidoscopy.

Between August 2006 and September 2015, 62,392 English UKFSST participants aged 60–74 were invited to gFOBT screening through the BCSP. This included 20,895 of 44,041 (47.4%) and 41,497 of 87,149 (47.6%) from the English UKFSST intervention and control groups, respectively (Figure 1). A lower proportion of those who refused FS (44.8%, 5532/12,337) were invited to gFOBT screening than those who accepted FS (48.5%, 15,363/31,704) or those in the control group. In comparison with the overall English UKFSST cohort of 131,190 UKFSST participants, those subsequently invited to gFOBT screening were younger (aged 57.8 vs. 60.0 at randomization) and a higher proportion (52.6% vs. 51.0%) were female (Tables 1 and 2).

**Table 2.** Characteristics of English UKFSST participants^a^ invited to gFOBT screening (*n* = 62,392).

	Control group	Intervention group		
		Overall	Screened by FS	Refused FS
Total invited to gFOBT screening – *n*	41,497	20,895	15,363	5532
Sex - *n* (%)				
Men	19,687 (47.4)	9943 (47.6)	7565 (49.2)	2378 (43.0)
Women	21,810 (52.6)	10,952 (52.4)	7798 (50.8)	3154 (57.0)
Age at randomization – years				
Median	57.8	57.8	57.7	57.8
Interquartile range	56.4–59.4	56.4–59.5	56.4–59.4	56.5–59.6
Age at first gFOBT invitation – years				
Median	69.0	69.0	69.0	69.0
Interquartile range	68.0–69.8	68.0–69.8	68.0–69.8	68.0–69.8
Interval between UKFSST randomization and first gFOBT invitation – years				
Median	10.9	10.9	11.0	10.9
Interquartile range	10.0–12.3	10.0–12.3	10.0–12.3	9.9–12.2
Range	8.0–19.1	7.9–18.5	7.9–18.5	8.3–18.1
Number of gFOBT invitations per person				
Mean	2.46	2.46	2.48	2.40
95% CI	2.45–2.48	2.44–2.48	2.46–2.50	2.36–2.44
Number of gFOBT screens per person				
Mean	1.73	1.71	1.88	1.23
95% CI	1.72–1.75	1.69–1.73	1.86–1.90	1.20–1.26

^a^After exclusions due to pre-randomization events and duplicates.

UKFSST: UK FS screening trial; FS: flexible sigmoidoscopy; gFOBT: guaiac faecal occult blood test.

UKFSST participants invited to gFOBT screening from the intervention group were similar, on average, to those invited from the control group (Table 2). In both groups, the median age at first gFOBT invitation was 69.0, just over half of invitees were women, a median of 10.9 years had elapsed between UKFSST randomization and first gFOBT invitation, and the mean number of gFOBT invitations sent over the study period was 2.46.

There were, however, differences among intervention group gFOBT invitees, between those who had refused and those who accepted FS screening (Table 2). A greater proportion of women refused FS than men, and for this reason a higher proportion of gFOBT invitees who had refused, rather than accepted FS, were women (57.0% vs. 50.8%). Furthermore, the average number of invitations sent and the average number of gFOBT screens were lower among those who had refused, rather than accepted, FS (2.40 vs. 2.48 invitations, and 1.23 vs. 1.88 screens, respectively).

Uptake of gFOBT screening was lower in the intervention group than in the control group at first invitation (65.7% vs. 67.6%, *p* < 0.01) and, to a lesser extent, at any invitation (74.9% vs. 75.8%, *p* = 0.01) (Table 3). Among intervention group gFOBT invitees, uptake was lower in those who had refused FS than in those who had accepted FS, at first invitation (47.7% vs. 72.2%) and at any invitation (58.3% vs. 80.9%). Uptake of gFOBT screening was marginally lower in men than women in all studied subgroups.

**Table 3.** Uptake of gFOBT screening in English UKFSST participants invited to gFOBT screening (*n* = 62,392).

	Control group (*n* = 41,497)		Intervention group (*n* = 20,895)		Difference		*p*	Intervention group – by uptake			
								Screened by FS (*n* = 15,363)		Refused FS (*n* = 5532)	
	*n*	%	*n*	%	%	95% CI		*n*	%	*n*	%
Screened by gFOBT (i.e. uptake) at first invitation											
Overall	28,041	67.6	13,730	65.7	1.9	1.1–2.6	<0.01	11,090	72.2	2640	47.7
Men	13,227	67.2	6502	65.4	1.8	0.7–2.9	<0.01	5408	71.5	1094	46.0
Women	14,814	67.9	7228	66.0	1.9	0.8–3.0	<0.01	5682	72.9	1546	49.0
Screened by gFOBT (i.e. uptake) at least once at any invitation											
Overall	31,451	75.8	15,649	74.9	0.9	0.2–1.6	0.01	12,424	80.9	3225	58.3
Men	14,836	75.4	7440	74.8	0.5	−0.5–1.6	0.32	6070	80.2	1370	57.6
Women	16,615	76.2	8209	74.9	1.2	0.2–2.2	0.02	6354	81.5	1855	58.8

UKFSST: UK FS screening trial; FS: flexible sigmoidoscopy; gFOBT: guaiac faecal occult blood test.

Of the 62,392 invited to gFOBT screening, 47,100 were screened by gFOBT at least once (Table 4). Test positivity was lower among intervention group than control group gFOBT screenees at first gFOBT screen (2.0% vs. 2.4%, *p* < 0.01) and at any gFOBT screen (4.4% vs. 5.0%, *p* < 0.01). Positivity at first gFOBT screen was higher in participants who had refused FS (2.8%) than in participants who had accepted (1.8%).

**Table 4.** Outcomes of gFOBT screening in English UKFSST participants who were screened by gFOBT at least once (*n* = 47,100).

	Control group (*n*=31,451)		Intervention group (*n*=15,649)		Difference		*p*	Intervention group – by uptake			
								Screened by FS (*n* = 12,424)		Refused FS (*n* = 3225)	
	*n*	%	*n*	%	%	95% CI		*n*	%	*n*	%
Positivity at first screen	768	2.4	319	2.0	0.4	0.1–0.7	<0.01	229	1.8	90	2.8
Yield of CRC at first gFOBT screen											
All sites	84	0.27	30^a^	0.19	0.08	−0.01–0.16	0.14	22	0.18	8	0.25
Proximal	23	0.07	10	0.06	0.01	−0.04–0.06	0.85	7	0.06	3	0.09
Distal	61	0.19	21	0.13	0.06	−0.02–0.13	0.16	16	0.13	5	0.16
Any positive gFOBT	1588	5.0	687	4.4	0.7	0.2–1.1	<0.01	523	4.2	164	5.1
Yield of CRC at any gFOBT screen											
All sites	181	0.58	57^a^	0.36	0.21	0.09–0.34	<0.01	42	0.34	15	0.47
Proximal	56	0.18	23	0.15	0.03	–0.04–0.11	0.48	18	0.15	5	0.16
Distal	125	0.40	35	0.22	0.17	0.07–0.28	<0.01	25	0.20	10	0.31

^a^One patient had both a distal and proximal lesion and therefore appears under yield of both distal and proximal cancer.

UKFSST: UK FS screening trial; FS: flexible sigmoidoscopy; gFOBT: guaiac faecal occult blood test.

The yield of CRC in those screened by gFOBT at least once was lower in intervention group than in control group gFOBT screenees, at first (0.19% vs. 0.27%, *p* = 0.14) and at any (0.36% vs. 0.58%, *p* < 0.01) gFOBT screen (Table 4). The yield of CRC in participants who had refused FS was higher (0.25% at first gFOBT screen, 0.47% at any gFOBT screen) than in those who had accepted FS (0.18% and 0.34%, respectively). The difference in yield of cancer between intervention and control group screenees was greater for distal cancer (0.22% vs. 0.40% at any gFOBT screen, *p* < 0.01) than for proximal cancer (0.15% vs. 0.18% at any gFOBT screen, *p* = 0.48). A greater proportion of cancers were later stage (Stage III/IV) among those diagnosed at gFOBT in the intervention group relative to the control group (36.8% vs. 27.6% of cancers diagnosed at any gFOBT screen), though this finding was not significant (*p* = 0.19; Supplemental Table 1). At first gFOBT screen, 1087 of 47,100 participants tested positive (Table 4) and 950 attended diagnostic investigation (Table 5). Among those attending diagnostic investigation from the intervention group and the control group, the PPVs for CRC were 10.3% (29/282) and 12.3% (82/668), respectively, and the PPVs for AAs were 22.7% (64/282) and 31.4% (210/668), respectively. The PPV for ACN was greatest in participants who had refused FS (46.2%, 36/78), but even among those who had received an FS, 27.9% (57/204) of those attending diagnostic investigation had ACN, and 10.3% (21/204) had CRC.

**Table 5.** Outcomes among those attending diagnostic investigation after positive gFOBT at first screen (*n* = 950).

	Control group (*n* = 668)		Intervention group (*n* = 282)		Difference		*p*	Intervention group – by uptake			
								Screened by FS (*n* = 204)		Refused FS (*n* = 78)	
	*n*	%	*n*	%	%	95% CI		*n*	%	*n*	%
PPV for CRC^a^											
All sites	82	12.3	29^b^	10.3	2.0	−2.3 – 6.3	0.44	21	10.3	8	10.3
Proximal	22	3.3	9	3.2	0.1	−2.4 – 2.6	1.00	6	2.9	3	3.9
Distal	60	9.0	21	7.4	1.5	−2.2 – 5.3	0.53	16	7.8	5	6.4
PPV for AA^c^											
All sites	210	31.4	64	22.7	8.7	2.7 – 14.8	<0.01	36	17.7	28	35.9
Proximal	67	10.0	19	6.7	3.3	−0.4 – 7.0	0.11	13	6.4	6	7.7
Distal	175	26.2	51	18.1	8.1	2.5–13.7	<0.01	26	12.8	25	32.1
PPV for ACN^d^											
All sites	292	43.7	93	33.0	10.7	4.1–17.4	<0.01	57	27.9	36	46.2
Proximal	89	13.3	28	9.9	3.4	−0.9 – 7.7	0.16	19	9.3	9	11.5
Distal	235	35.2	72	25.5	9.6	3.4–15.9	<0.01	42	20.6	30	38.5

^a^Positive predictive value among participants attending diagnostic investigation. Only cancers identified through the BCSP are included in these figures.

^b^One patient had both a distal and proximal lesion and therefore appears under PPV of both distal and proximal cancer.

^c^Advanced adenomas. Only includes advanced adenomas where it was the most advanced finding (i.e. in cases where colorectal cancer was not found).

^d^Advanced colorectal neoplasia: colorectal cancer or advanced adenomas.

FS: flexible sigmoidoscopy; gFOBT: guaiac faecal occult blood test; PPV: positive predictive value; AA: advanced adenoma; CRC: colorectal cancer; CAN: advanced colorectal neoplasia.

The PPV for distal cancer at first gFOBT screen was lower in the intervention group (7.4%, 21/282) than in the control group (9.0%, 60/668) diagnostic investigation attendees (*p* = 0.53). The PPVs for proximal cancer were very similar in the two groups (3.2% vs. 3.3%, *p* = 1.0). The PPV for distal ACN was lower in intervention group than in control group attendees (25.5% vs. 35.2%, *p* < 0.01), as was the PPV for proximal ACN (9.9% vs. 13.3%, *p* = 0.16). Among intervention group, participants who had refused FS, the PPV for distal ACN was 38.5% (30/78), and the PPV for proximal ACN was 11.5% (9/78). Among participants who had received FS, the PPV for distal ACN was 20.6% (42/204), and the PPV for proximal ACN was 9.3% (19/204).

## Discussion

At gFOBT, test positivity and yield of CRC were slightly lower among those who had previously been offered, or had received, an FS. One potential explanation for this is that prior FS, through the removal of adenomas, reduced the number of CRCs and adenomas that could be found by gFOBT screening. Reduced incidence of CRC in the UKFSST intervention group at 17 years post-randomization, as reported elsewhere, supports this explanation.^17^ Furthermore, as would be expected given that FS is a screening examination of the distal colon and rectum, the difference in yield of gFOBT screening in the UKFSST intervention group versus the control group was greater for distal than for proximal cancer.

Though the yield of CRC was lower among UKFSST intervention group gFOBT screenees, gFOBT screening after FS could still be of potential benefit. However, given the effectiveness of FS at reducing CRC incidence for at least 17 years after screening, and given minimal randomized trial evidence that gFOBT or FIT reduce incidence, it is likely that extensive faecal testing would be required to achieve a benefit similar to that accomplished already by once-only FS.^7,17^

Achieving high uptake is crucial to a successful screening programme. Uptake of gFOBT at first invite was lower in participants previously offered FS (65.7%) than in those allocated to the control group (67.6%) in the UKFSST. While uptake was lower, the absolute difference was only 1.9%. Furthermore, in participants who had been offered and had accepted FS, uptake of gFOBT screening was high (72.2%). In general, uptake was considerably higher than typically observed in the BCSP, but this is to be expected given that only individuals who expressed an interest in screening were eligible for the UKFSST.^1,21^

Though uptake was lower than in other groups, many participants who had refused FS were screened by gFOBT (uptake of 47.7% at first, and 58.3% at any, gFOBT invitation). A number of researchers have examined the uptake of FIT in FS non-responders. Hol et al.^22^ offered FIT to 4407 FS screening non-attenders and found that 25% attended FIT screening. Similarly, Senore et al.^23^ found that among 37,691 FS screening non-responders, uptake of FIT offered six months following non-response was 19.3%. Subsequent faecal occult blood testing (either gFOBT or FIT) could be used to increase screening uptake above that observed with FS alone.

The differences identified between participants who had refused and those who had accepted FS may partly reflect the impact of FS, but may also be due to FS refusers being a less healthy group (i.e. healthy user bias).^24^ Survival was lower, and PPV for ACN from gFOBT screening was higher, in the FS refusers than in the control group.

Though the sample size of our study is relatively large, only a subset of participants tested gFOBT positive, and only a subset of these had an outcome of interest, such as CRC. The low frequency of events limited statistical power, particularly for PPVs and yield.

Many UKFSST participants were not invited to gFOBT screening. Reasons for non-invitation include mortality, emigration, and being over the eligible age limit. Differential CRC incidence and mortality due to FS may have affected, to some extent, the composition of the intervention and control group invited to gFOBT screening. However, at gFOBT invitation, the two groups were broadly similar on many characteristics, including age and sex.

Generalizability of our findings to the current English BCSP is weakened by patient and screening characteristics unique to the study cohort. Only individuals who expressed an interest in screening were eligible for UKFSST, and for this reason we would have expected higher uptake of biennial gFOBT in this group than in the national BCSP or in other studies. The average interval between gFOBT screening and FS was 10.9 years. This is considerably longer than the current 5-year interval in the BCSP, where FS is being offered at age 55 and gFOBT screening from age 60. This longer interval means that there was more time between screenings for ACN to develop. UKFSST subjects invited to gFOBT screening were older than average BCSP invitees, with a median age at first gFOBT invitation of 69.0. Prevalence of ACN is known to increase with age.^25^

While gFOBT has been the test used in the English BCSP, a different stool test, FIT, is being introduced in 2018. Both gFOBT and FIT are mailed tests for occult blood in faeces. At low faecal haemoglobin positivity thresholds FIT is more sensitive than gFOBT, and uptake of FIT is expected to be slightly higher than gFOBT.^26^

Though there are a number of limitations, our study also has considerable strengths. Randomization applied in the UKFSST means that, except for differential attrition, at gFOBT invitation the intervention and control groups should be similar. Another strength of the study is that by using cancer registry data, we have been able to check for cancers diagnosed outside the BCSP diagnosed shortly after a positive gFOBT.

The findings of this study indicate that the combination of gFOBT screening and FS could be beneficial in terms of screening uptake, yield of CRC, and yield of AAs. FS is highly sensitive for neoplasia located in the distal colon and rectum, more so than gFOBT or FIT.^9,27–29^ However, the ability of FS screening to detect cancer or adenomas in the proximal colon is limited and depends on the criteria determining referral for colonoscopy.^30^ There is evidence to suggest that the performance of gFOBT and FIT is also limited in the proximal colon, with lower sensitivity than for findings in the distal colon and rectum.^31^ Given the apparent limitations of both techniques in detecting proximal findings, there remains a need for a low cost non-invasive screening test that is highly sensitive for proximal colon cancer.

## Conclusion

Uptake, positivity, PPV and yield of gFOBT screening were lower among UKFSST participants who had been offered FS than in those who had not. Among FS screened participants attending diagnostic investigation following a positive gFOBT, ACN was found in just over a quarter of individuals. The findings of this study indicate that, dependent on cost-effectiveness, a combination of FS and faecal occult blood test screening might be beneficial.

## Supplemental Material

Supplemental material for Effect of once-only flexible sigmoidoscopy screening on the outcomes of subsequent faecal occult blood test screening Supplemental material for Effect of once-only flexible sigmoidoscopy screening on the outcomes of subsequent faecal occult blood test screening by Jeremy P Brown, Kate Wooldrage, Ines Kralj-Hans, Suzanne Wright, Amanda J Cross and Wendy S Atkin in Journal of Medical Screening

## References

[1] Logan RFA Patnick J Nickerson C et al . Outcomes of the Bowel Cancer Screening Programme (BCSP) in England after the first 1 million tests. Gut 2012; 61: 1439–1446. 22156981 10.1136/gutjnl-2011-300843 PMC3437782

[2] Geurts S Massat N and Duffy S. Likely effect of adding flexible sigmoidoscopy to the English NHS Bowel Cancer Screening Programme: impact on colorectal cancer cases and deaths. Br J Cancer 2015; 113: 142–149. 26110973 10.1038/bjc.2015.76 PMC4647530

[3] Atkin WS Edwards R Kralj-Hans I et al . Once-only flexible sigmoidoscopy screening in prevention of colorectal cancer: a multicentre randomised controlled trial. Lancet 2010; 375: 1624–1633. 20430429 10.1016/S0140-6736(10)60551-X

[4] Holme Ø Løberg M Kalager M et al . Effect of flexible sigmoidoscopy screening on colorectal cancer incidence and mortality: a randomized clinical trial. JAMA 2014; 312: 606–615. 25117129 10.1001/jama.2014.8266 PMC4495882

[5] Schoen RE Pinsky PF Weissfeld JL et al . Colorectal-cancer incidence and mortality with screening flexible sigmoidoscopy. N Engl J Med 2012; 366: 2345–2357. 22612596 10.1056/NEJMoa1114635 PMC3641846

[6] Segnan N Armaroli P Bonelli L et al . Once-only sigmoidoscopy in colorectal cancer screening: follow-up findings of the Italian Randomized Controlled Trial–SCORE. J Natl Cancer Inst 2011; 103: 1310–1322. 21852264 10.1093/jnci/djr284

[7] Hewitson P Glasziou P Watson E et al . Cochrane systematic review of colorectal cancer screening using the fecal occult blood test (hemoccult): an update. Am J Gastroenterology 2008; 103: 1541–1549. 10.1111/j.1572-0241.2008.01875.x 18479499

[8] Benson VS Atkin WS Green J et al . Toward standardizing and reporting colorectal cancer screening indicators on an international level: the international colorectal cancer screening network. Int J Cancer 2012; 130: 2961–2973. 21792895 10.1002/ijc.26310

[9] Rozen P Ron E Fireman Z et al . The relative value of fecal occult blood tests and flexible sigmoidoscopy in screening for large bowel neoplasia. Cancer 1987; 60: 2553–2558. 3664435 10.1002/1097-0142(19871115)60:10<2553::aid-cncr2820601034>3.0.co;2-s

[10] Berry DP Clarke P Hardcastle JD et al . Randomized trial of the addition of flexible sigmoidoscopy to faecal occult blood testing for colorectal neoplasia population screening. Br J Surg 1997; 84: 1274–1276. 9313712

[11] Rasmussen M Kronborg O Fenger C et al . Possible advantages and drawbacks of adding flexible sigmoidoscopy to hemoccult-II in screening for colorectal cancer. A randomized study. Scand J Gastroenterol 1999; 34: 73–78. 10048736 10.1080/00365529950172862

[12] Segnan N Senore C Andreoni B et al . Randomized trial of different screening strategies for colorectal cancer: patient response and detection rates. J Natl Cancer Inst 2005; 97: 347–357. 15741571 10.1093/jnci/dji050

[13] Denis B Gendre I Aman F et al . Colorectal cancer screening with the addition of flexible sigmoidoscopy to guaiac-based faecal occult blood testing: a French population-based controlled study (Wintzenheim trial). Eur J Cancer 2009; 45: 3282–3290. 19665368 10.1016/j.ejca.2009.06.015

[14] Hol L van Leerdam ME van Ballegooijen M et al . Screening for colorectal cancer: randomised trial comparing guaiac-based and immunochemical faecal occult blood testing and flexible sigmoidoscopy. Gut 2010; 59: 62–68. 19671542 10.1136/gut.2009.177089

[15] Randel KR Schult AL Botteri E et al . Mo1946 bowel cancer screening in Norway (BCSN) – a randomized pilot study comparing flexible sigmoidoscopy (FS) to fecal immunochemical test (FIT). Gastroenterology 2015; 148: S747–S747.

[16] Holme O Bretthauer M Fretheim A et al . Flexible sigmoidoscopy versus faecal occult blood testing for colorectal cancer screening in asymptomatic individuals. Cochrane Database Syst Rev 2013; 9: CD009259. 10.1002/14651858.CD009259.pub2 PMC9365065 24085634

[17] Atkin W Wooldrage K Parkin DM et al . Long term effects of once-only flexible sigmoidoscopy screening after 17 years of follow-up: the UK flexible sigmoidoscopy screening randomised controlled trial. Lancet 2017; 389: 1299–1311. 28236467 10.1016/S0140-6736(17)30396-3 PMC6168937

[18] McGregor LM Bonello B Kerrison RS et al . Uptake of bowel scope (flexible sigmoidoscopy) screening in the English national programme: the first 14 months. J Med Screen 2016; 23: 77–82. 26387824 10.1177/0969141315604659

[19] Atkin WS Cook CF Cuzick J et al . Single flexible sigmoidoscopy screening to prevent colorectal cancer: baseline findings of a UK multicentre randomised trial. Lancet 2002; 359: 1291–1300. 11965274 10.1016/S0140-6736(02)08268-5

[20] Cairns SR Scholefield JH Steele RJ et al . Guidelines for colorectal cancer screening and surveillance in moderate and high risk groups (update from 2002). Gut 2010; 59: 666–689. 20427401 10.1136/gut.2009.179804

[21] Lo SH Halloran S Snowball J et al . Colorectal cancer screening uptake over three biennial invitation rounds in the English Bowel Cancer Screening Programme. Gut 2015; 64: 282–291. 24812001 10.1136/gutjnl-2013-306144 PMC4316922

[22] Hol L Kuipers EJ van Ballegooijen M et al . Uptake of faecal immunochemical test screening among nonparticipants in a flexible sigmoidoscopy screening programme. Int J Cancer 2012; 130: 2096–2102. 21702046 10.1002/ijc.26260

[23] Senore C Ederle A Benazzato L et al . Offering people a choice for colorectal cancer screening. Gut 2013; 62: 735–740. 22442162 10.1136/gutjnl-2011-301013

[24] Shrank WH Patrick AR Brookhart MA. Healthy user and related biases in observational studies of preventive interventions: a primer for physicians. J Gen Intern Med 2011; 26: 546–550. 21203857 10.1007/s11606-010-1609-1 PMC3077477

[25] Brenner H Altenhofen L and Hoffmeister M. Sex, age, and birth cohort effects in colorectal neoplasms: a cohort analysis. Ann Intern Med 2010; 152: 697–703. 20513827 10.7326/0003-4819-152-11-201006010-00002

[26] Moss S Mathews C Day T et al . Increased uptake and improved outcomes of bowel cancer screening with a faecal immunochemical test: results from a pilot study within the national screening programme in England. Gut 2017; 66: 1631–1644. 27267903 10.1136/gutjnl-2015-310691

[27] Brevinge H Lindholm E Buntzen S et al . Screening for colorectal neoplasia with faecal occult blood testing compared with flexible sigmoidoscopy directly in a 55-56 years' old population. Int J Colorectal Dis 1997; 12: 291–295. 9401844 10.1007/s003840050108

[28] Cheng TI Wong JM Hong CF et al . Colorectal cancer screening in asymptomatic adults: comparison of colonoscopy, sigmoidoscopy and fecal occult blood tests. J Formosan Med Assoc 2002; 101: 685–690. 12517041

[29] Khalid-de Bakker CA Jonkers DM Sanduleanu S et al . Test performance of immunologic fecal occult blood testing and sigmoidoscopy compared with primary colonoscopy screening for colorectal advanced adenomas. Cancer Prev Res 2011; 4: 1563–1571. 10.1158/1940-6207.CAPR-11-0076 21750209

[30] Castro I Estevez P Cubiella J et al . Diagnostic performance of fecal immunochemical test and sigmoidoscopy for advanced right-sided colorectal neoplasms. Dig Dis Sci 2015; 60: 1424–1432. 25407805 10.1007/s10620-014-3434-6

[31] Hirai HW Tsoi KK Chan JY et al . Systematic review with meta-analysis: faecal occult blood tests show lower colorectal cancer detection rates in the proximal colon in colonoscopy-verified diagnostic studies. Aliment Pharmacol Ther 2016; 43: 755–764. 26858128 10.1111/apt.13556

