# Supplementary material for: Effect of once-only flexible sigmoidoscopy screening on the outcomes of subsequent faecal occult blood test screening
Source: J Med Screen. 2018 Oct 3;26(1):11–8. doi: 10.1177/0969141318785654 (PMC6376653; doi:10.1177/0969141318785654)
Supplement: Supplemental material for Effect of once-only flexible sigmoidoscopy screening on the outcomes of subsequent faecal occult blood test screening [file Supplemental_material.pdf]

Supplementary material

Supplementary Table 1: Stage distribution of colorectal cancers detected through gFOBT screening

	Control group		Intervention group					
			Overall		Screened by FS		Refused FS	
	n	%	n	%	n	%	n	%
**CRC at first gFOBT screen**	**84**		**30**		**22**		**8**	
Stage								
I	37	44.0	7	23.3	6	27.3	1	12.5
II	10	11.9	6	20.0	4	18.2	2	25.0
III/IV	23	27.4	12	40.0	8	36.4	4	50.0
Not available	14	16.7	5	16.7	4	18.2	1	12.5
**CRC at any gFOBT screen**	**181**		**57**		**42**		**15**	
Stage								
I	65	35.9	16	28.1	14	33.3	2	13.3
II	30	16.6	10	17.5	8	19.1	2	13.3
III/IV	50	27.6	21	36.8	14	33.3	7	46.7
Not available	36	19.9	10	17.5	6	14.3	4	26.7

Supplementary Table 2: Outcomes of gFOBT screening in UKFSST participants who were screened by gFOBT at least once stratified by sex (n=47,100)

	Control group (n=31,451)		Intervention group (n=15,649)		Difference		p-value	Intervention group – by uptake			
								Screened by FS (n=12,424)		Refused FS (n=3,225)	
	n	%	n	%	%	95% CI		n	%	n	%
**Men**	14,836		7,440					6,070		1,370	
Positivity at first screen	452	3.0	185	2.5	0.56	0.1-1.0	0.02	140	2.3	45	3.3
Yield of CRC at first gFOBT screen											
All sites	50	0.34	22*	0.30	0.04	-0.1-0.2	0.71	17	0.28	5	0.36
Proximal	16	0.11	8	0.11	0.00	-0.1-0.1	1.0	6	0.10	2	0.15
Distal	34	0.23	15	0.20	0.03	-0.1-0.2		12	0.20	3	0.22
Any positive gFOBT	928	6.3	384	5.2	1.09	0.5-1.7	<0.01	303	5.0	81	5.9
Yield of CRC at any gFOBT screen											
All sites	114	0.77	38*	0.51	0.26	0.0-4.7	0.03	30	0.49	8	0.58
Proximal	35	0.24	16	0.22	0.02	-0.1-0.2	0.88	13	0.21	3	0.22
Distal	79	0.53	23	0.31	0.22	0.1-0.3	0.02	18	0.30	5	0.36
**Women**	16,615		8,209					6,354		1,855	
Positivity at first screen	316	1.9	134	1.6	0.27	-0.1-0.6	0.14	89	1.4	45	2.4
Yield of CRC at first gFOBT screen											
All sites	34	0.20	8	0.10	0.11	0.0-0.2	0.07	5	0.08	3	0.16
Proximal	7	0.04	2	0.02	0.02	0.0-0.1	0.73	1	0.02	1	0.05
Distal	27	0.16	6	0.07	0.09	0.0-0.2	0.09	4	0.06	2	0.11
Any positive gFOBT	660	4.0	303	3.7	0.28	-0.2-0.8	0.29	220	3.5	83	4.5
Yield of CRC at any gFOBT screen											
All sites	67	0.40	19	0.23	0.17	0.0-0.3	0.03	12	0.19	7	0.38
Proximal	21	0.13	7	0.09	0.04	0.0-0.1	0.43	5	0.08	2	0.11
Distal	46	0.28	12	0.15	0.13	0.0-0.2	0.05	7	0.11	5	0.27

***** One patient had both a distal and proximal lesion and therefore appear under yield of both distal and proximal cancer.

Supplementary Table 3: Outcomes among those attending diagnostic investigation after positive gFOBT at first screen stratified by sex (n=950)

	Control group (n=668)		Intervention group (n=282)		Difference		p-value	Screened by FS (n=204)		Refused FS (n=78)	
	n	%	n	%	%	95% CI		n	%	n	%
**Men**	398		170					127		43	
PPV for CRC*											
All sites	48	12.1	21§	12.4	-0.3	-6.2-5.6	1.0	16	12.6	5	11.6
Proximal	15	3.8	7	4.1	-0.3	-3.9-3.2	0.82	5	3.9	2	4.7
Distal	33	8.3	15	8.8	-0.5	-5.6-4.5		12	9.4	3	7.0
PPV for AA†											
All sites	144	36.2	45	26.5	9.7	1.5-17.9	0.03	24	18.9	21	48.8
Proximal	54	13.6	12	7.1	6.5	1.4-11.6	0.03	8	6.3	4	9.3
Distal	118	29.6	38	22.4	7.3	-0.4-15.0	0.08	19	15.0	19	44.2
PPV for ACN‡											
All sites	192	48.2	66	38.8	9.4	0.6-18.2	0.04	40	31.5	26	60.5
Proximal	69	17.3	19	11.2	6.2	0.1-12.2	0.08	13	10.2	6	14.0
Distal	151	37.9	53	31.2	6.8	-1.7-15.2	0.13	31	24.4	22	51.2
**Women**	270		112					77		35	
PPV for CRC*											
All sites -	34	12.6	8	7.1	5.4	-0.7-11.6	0.15	5	6.5	3	8.6
Proximal	7	2.6	2	1.8	0.8	-2.3-3.9	1.0	1	1.3	1	2.9
Distal	27	10.0	6	5.4	4.6	-0.8-10.1	0.16	4	5.2	2	5.7
PPV for AA†											
All sites	66	24.4	19	17.0	7.5	-1.2-16.1	0.14	12	15.6	7	20.0
Proximal	13	4.8	7	6.2	-1.4	-6.6-3.7	0.62	5	6.5	2	5.7
Distal	57	21.1	13	11.6	9.5	2.8-17.2	0.03	7	9.1	6	17.1
PPV for ACN‡											
All sites	100	37.0	27	24.1	12.9	3.1-22.7	0.02	17	22.1	10	28.6
Proximal	20	7.4	9	8.0	-0.6	-6.6-5.3	0.83	6	7.8	3	8.6
Distal	84	31.1	19	17.0	14.1	5.3-23.0	<0.01	11	14.3	8	22.9

* Positive predictive value among participants attending diagnostic investigation. Only cancers identified through the BCSP are included in these figures.

† Advanced adenomas. Only includes advanced adenomas where it was the most advanced finding (i.e. in cases where colorectal cancer was not found).

‡ Advanced colorectal neoplasia: colorectal cancer or advanced adenomas.

§ One patient had both a distal and proximal lesion and therefore appear under PPV of both distal and proximal cancer.
